# Hybrid Deep Learning Model for Endoscopic Lesion Detection and Classification Using Endoscopy Videos

**DOI:** 10.3390/diagnostics12010043

**Published:** 2021-12-26

**Authors:** M Shahbaz Ayyaz, Muhammad Ikram Ullah Lali, Mubbashar Hussain, Hafiz Tayyab Rauf, Bader Alouffi, Hashem Alyami, Shahbaz Wasti

**Affiliations:** 1 Department of Computer Science, University of Gujrat, Gujrat 50700, Pakistan; shahbazayyaz2@gmail.com (M.S.A.); mubbashar.hussain@uog.edu.pk (M.H.); 2 Department of Information Sciences, University of Education Lahore, Lahore 41000, Pakistan; m.i.lali@ue.edu.pk (M.I.U.L.); shahbazwasti@ue.edu.pk (S.W.); 3 Centre for Smart Systems, AI and Cybersecurity, Staffordshire University, Stoke-on-Trent ST4 2DE, UK; 4 Department of Computer Science, College of Computers and Information Technology, Taif University, P. O. Box 11099, Taif 21944, Saudi Arabia; balouffi@tu.edu.sa (B.A.); hyami@tu.edu.sa (H.A.)

**Keywords:** stomach diseases, deep learning, endoscopy videos, genetic algorithm

## Abstract

In medical imaging, the detection and classification of stomach diseases are challenging due to the resemblance of different symptoms, image contrast, and complex background. Computer-aided diagnosis (CAD) plays a vital role in the medical imaging field, allowing accurate results to be obtained in minimal time. This article proposes a new hybrid method to detect and classify stomach diseases using endoscopy videos. The proposed methodology comprises seven significant steps: data acquisition, preprocessing of data, transfer learning of deep models, feature extraction, feature selection, hybridization, and classification. We selected two different CNN models (VGG19 and Alexnet) to extract features. We applied transfer learning techniques before using them as feature extractors. We used a genetic algorithm (GA) in feature selection, due to its adaptive nature. We fused selected features of both models using a serial-based approach. Finally, the best features were provided to multiple machine learning classifiers for detection and classification. The proposed approach was evaluated on a personally collected dataset of five classes, including gastritis, ulcer, esophagitis, bleeding, and healthy. We observed that the proposed technique performed superbly on Cubic SVM with 99.8% accuracy. For the authenticity of the proposed technique, we considered these statistical measures: classification accuracy, recall, precision, False Negative Rate (FNR), Area Under the Curve (AUC), and time. In addition, we provided a fair state-of-the-art comparison of our proposed technique with existing techniques that proves its worthiness.

## 1. Introduction

The stomach is a muscular organ that helps to digest food. It is located on the left side of the upper abdomen. It takes food from the esophagus through a muscular valve called the lower esophageal sphincter [1]. The stomach performs three tasks. Firstly, it stores the food that we eat. Secondly, it mixes the food and discharges gastric juices that help break down and digest food. Then it moves that mixture into the small intestine. Sometimes, it can be affected by different diseases, such as gastritis, gastroparesis, diarrhea, peptic ulcers, Crohn’s disease, etc. Gastritis is the swelling of the stomach lining. Intense gastritis may come on abruptly. Chronic gastritis happens gradually. As indicated by the Cleveland Clinic, 8 out of 1000 individuals have intense gastritis, and 2 out of 10,000 have chronic gastritis. The symptoms of gastritis are vomiting, hiccups, indigestion, etc. The significant causes behind gastritis are stress, viral infection, etc. A peptic ulcer is another critical disease of the stomach. If the internal lining of the stomach is destroyed, then the patients will likely be positive for a stomach disease. Most are situated in the top layer of the inward covering. An ulcer that goes entirely through the stomach lining is a hole and requires prompt clinical consideration. Fatigue, vomiting, and feeling hungry soon after eating are the symptoms of a peptic ulcer. If the disease is not diagnosed in the early stages, it can convert into cancer. One of the fatal stomach cancers is gastric cancer. It is the fourth most common cancer, as well as it is the fourth primary cause of death for cancer patients worldwide [2].

According to a survey on stomach cancer, 26% of men and 11% of women have this type of cancer globally. In men, stomach cancer is ranked second below lung cancer, and in women, it is ranked third below lung and breast cancer [3]. Generally, stomach cancer starts with an ulcer and gastritis-type complaints. It can affect the lymph nodes and peripheral organs. Only healthy food and early detection of infection can help to overcome this disease. 

In 2012, more than 951,000 cases of gastric cancer were diagnosed, which was 7% of the total cancers [4]. Thereafter, more than 950,000 patients with gastric cancer are being diagnosed every year [2]. According to the American Cancer Society’s 2019 report on stomach cancer in the United States (US), about 27,510 cases of stomach cancer were diagnosed, of which 17230 were men and the remaining cases were women. About 11,140 died due to this cancer (6800 women and 4340 men). According to the World Health Organization’s 2018 report on stomach cancer in Pakistan, about 4154 cases of stomach cancer were diagnosed, and about 3923 died due to stomach cancer. This means that approximately 95% of patients with stomach cancer died. These stats are alarming. From the past until 1930, stomach cancer was the prominent cause of death in the US. Over the last ten years, stomach cancer’s decreasing percentage has been 1.5. The reasons for the decline were the use of fresh vegetables and fruits and the use of a proper antibiotic that kills helicobacter pylori (H pylori) bacteria [5]. Another reason was the early detection of stomach diseases.

Deep-learning-based techniques are playing a vital role in the production of authentic and more detailed results of stomach diseases. The results obtained from the computer vision or deep-learning-based approaches are more accurate than manually obtained results. As stomach diseases are increasing day by day, it is very important to develop a reliable system that automatically detects stomach diseases at an early stage. Image-enhanced endoscopy (IEE) is an excellent method to detect gastrointestinal neoplasm, such as narrowband imaging (NBI) with magnifying endoscopy, which is very beneficial for the detection of esophageal and pharyngeal cancers. However, IEE efficiency is not effective for detecting gastric cancer due to its low brightness. Moreover, gastric cancer is hard to detect because of the background mucosal change in gastritis [6]. In other techniques, computerized tomography (CT) scans and a special type of X-ray exam called a barium swallow are also present. Furthermore, there are also various computer-aided design (CAD) systems present which help us to diagnose different stomach diseases in the early stages using endoscopic videos or images. Endoscopy is a procedure that allows a surgeon to look inside a human’s body without making any incisions. There are two types of endoscopy. The first is simple endoscopy and the second is capsule endoscopy.

**Figure 1:**
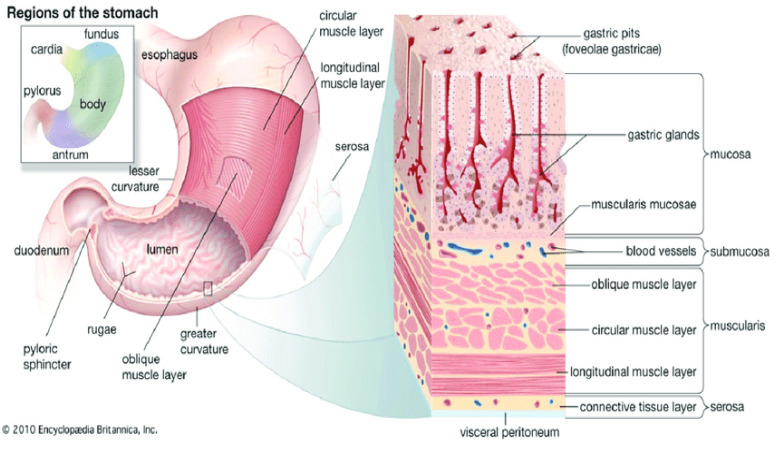
Structure of the human stomach and gastric wall [7].

The structure of a human stomach is discussed in detail by [7] and is illustrated in Figure 1. 

The early detection of diseases helps greatly to reduce the death rate. For early detection, we need a CAD system that helps us to detect healthy and unhealthy tissues accurately because the detection of diseases from the endoscopy video is time-consuming and hectic for doctors due to a large number of frames in the video. Selection of the frames containing abnormalities is also a difficult task because typically only 5% of the frames contain abnormalities [8]. Several researchers [3,6,9,10,11,12,13,14,15,16] have suggested different solutions for the automated detection of stomach diseases. However, due to the resemblance of different symptoms including color, shape, texture, etc., it is challenging to accurately classify the type of infection. Most of the previous work was conducted on the detection of a single disease/infection [1,11,16,17,18]. Accurate classification of four significant diseases (gastritis, esophagitis, peptic ulcers, and bleeding) and healthy images using a single framework is still challenging. That is why we need an automatic system that can classify healthy and unhealthy tissues with more accuracy. In this paper, we proposed a methodology for detecting and classifying four major stomach diseases. The proposed approach for stomach disease detection:

1. The proposed methodology comprises seven significant steps: data acquisition, preprocessing of data, transfer learning of deep models, feature extraction, feature selection, hybridization, and classification.
2. We selected two different CNN models (VGG19 and Alexnet) to extract features and then used transfer learning on the feature vectors before using them as feature extractors. In feature selection, heuristic GA was used to select rich information from the extracted feature vectors.
3. The fusion was performed on the selected features of both models using a serial-based approach. Finally, the best features were provided to multiple machine learning classifiers for detection and classification.

## 2. Related Work

Computer-aided diagnosis (CAD) plays a vital role in medical imaging, allowing accurate results to be obtained in minimal time [13,19,20,21]. Various computer techniques are being used in medical imaging for the automatic detection of diseases. Artificial intelligence (AI) and its applications such as machine learning and deep learning are the best examples [13,19,22,23]. 

Machine learning is a technique in which a computer takes help from data to improve its performance without explicit instructions [19]. Supervised and unsupervised learning are two types of machine learning. Machine learning demands labeled data at the start, and it takes time to tune the features [24]. On the other hand, deep learning uses neural networks that can automatically select the features by assigning weights to neurons. In 2006, deep learning came as a new and upgraded face of machine learning [25]. Deep learning is used in many fields, such as computer vision, natural language processing, bioinformatics, and medical imaging [26]. In the last few years, it changed every bit of technology. It allows the machine to recognize images and voices by identifying their patterns. The main goal is that now the machine can learn quickly without providing special attention to data [25]. 

In the recent past, many researchers used machine learning and deep learning to perform clinical tasks, such as analyzing a massive volume of unstructured data, processing images, identifying diseases, etc [3,12,19,23,27]. Medical imaging gained much attention from researchers, due to its importance in the world. Disease detection and its classification are two very hot topics in medical imaging. There are many segmentation and classification methods based on machine learning and computer-based techniques that are used in medical imaging. Whereas stomach disease detection is a favorite among all researchers, there are various stomach disease detection, segmentation, and classification methods that are already implemented, and every method consists of various steps, such as noise removal [28], segmentation [29], extraction of features [1,30], feature selection, feature fusion [31], and classification [1,15,27].

To improve the quality of frames/images, preprocessing is a mandatory step. Some researchers use narrow-band imaging (NBI) and chromo-endoscopy using indigo carmine spraying [2,6,10]. Interestingly, some researchers follow the pre-step before preprocessing, such as [10], who used the inclusion and exclusion criteria for images before preprocessing. Included images needed to have standard white-light and narrow-band imaging and images that needed to be magnified or had poor quality were excluded. Based on these criteria, they selected 13,584 images of gastric cancer from 69 consecutive patients with 77 gastric cancer lesions. The authors in [3] used a gradient-based technique to detect the edges of the images. This authors first changed the images from color to grayscale, then applied the derivative mask on the grayscale images to obtain the horizontal (*f_a_*) and vertical (*f_b_*) gradient separately. The formulas are shown in Equations (1) and (2).

(1) $$f_{a}\;\left(a,\;b\right)=I\;\left(a+1,\;b\right)\;-\;I\;\left(a-1,\;b\right)$$

(2) $$f_{b}\;\left(a,\;b\right)=I\;\left(a,\;b+1\right)\;-\;I\;\left(a,\;b-1\right)$$

For the segmentation of images, some researchers used region growing (RG), statistical region merging (SRM), and statistical region merging with region growing (SRMWRG) techniques [13]. In RG, the regions should have smooth boundaries and a single pixel can not belong to more than one region. The RG method produced a good result as compared to the other two. SRMWRG also showed good results, but they were not good as the RG results. On the other hand, SRM produced the worst results when compared to the other techniques.

Scholars widely use machine learning and deep learning techniques to develop models for detecting stomach diseases. The authors in [17] developed a system to detect an ulcer using two descriptors, the complete local binary pattern (CLBP) and the global local oriented edge magnitude pattern (Global LOEMP), to obtain texture features and color features. The accuracy of the proposed method (94.07% using SVM) was high compared with the other existing methods at that time. Feature extraction is an important step in computer-aided diagnosis systems. It plays a significant role in obtaining better results from the stomach disease detection process. Several researchers used different techniques for the extraction of features, such as discrete wavelet transform (DWT) [32], the complete local binary pattern (CLBP), and color Global LOEMP [17]. Feature dimension reduction is conducted by cropping the image to obtain a closer look at the infected area [33,34]. In [33], the authors reduced the dimensions of features into lower dimensions using DFT-NCA methods. 

Classification of frames/images into healthy and unhealthy parts is the most critical step of a system. Computer-aided diagnosis systems use different machine learning and deep learning classifiers to classify images. There are different classifiers, such as support vector machine (SVM), decision tree (DT), convolutional neural network (CNN), k-nearest neighbors (KNN), discrete Fourier transforms (DFT), naïve Bayes(NB), artificial neural network (ANN), random forest (RF), and discriminant analysis (LDA). In [33], the authors used the naïve Bayes classifier in an extension of his previous work that produced 90.27% accurate results in their proposed systems. In [35], the authors used different classifiers such as KNN, SVM, and DT for the detection of gastric deviations. SVM outperformed the other classifiers with an accuracy of 87.2%. 

The convolutional neural network (CNN) used by [6,10,12] gave 92.2%, 86.7%, and 97.25% accuracy, respectively. CNN is a deep learning model that consists of multiple layers. The deep convolutional neural network (DCNN) was used by [25] for the classification of gastric cancer images. The dataset consisted of over 3000 images and the DCNN produced excellent results, with an accuracy of 96.88%. The ANN and RF were used by [22] in their proposed model that gave 96.26% accuracy for the classification of stomach cancer images. A machine learning classifier SVM was used by multiple researchers for the classification of stomach diseases [4,17,21]. The obtained results were 96.07%, 96.3%, and 98.95%, respectively. In their proposed methodology of ulcer detection, [36] used VGG16 for features extraction and SVM for classification purposes. This combination provided 98.4% accuracy on their privately collected dataset.

BPNN is another technique that is widely used by researchers for the detection and classification of stomach diseases. The authors in [1] used BPNN for a stomach disorder and [18] used the same method for Crohn’s disease detection. They acheived 87.5% and 97.67% accuracy, respectively. In [29], the authors applied ResNet and LSTM methods to a huge dataset that consists of more than 50,000 images for multiple disease detection. ResNet and LSTM produced 97.05% accuracy, which was excellent for such a vast dataset. The previous work is discussed in detail in Table 1, below.

## 3. Materials and Methods

The proposed methodology consists of seven steps, such as data acquisition, preprocessing, feature extraction using VGG19 and Alexnet, transfer learning, feature selection using a genetic algorithm, feature fusion, and classification. In the first step, we collected a dataset from a medical specialist. The dataset was in rough form. We normalized our collected data by taking the input of a gastrologist. In the second step, we applied different filters to our collected dataset, now available in images. After that, we selected two convolutional neural networks, named Alexnet and VGG19, to extract features. The best thing about these models was that there was no need to develop a system from scratch, as both models were already trained on thousands of Imagenet datasets and could be classified into 1000 different classes. We performed transfer learning on these models that helped in the modification process. After transfer learning, our models were modified and ready to extract features from our dataset. 

We used the genetic algorithm for feature selection purposes. As we extracted features with the two models, we needed to select features separately. One feature at a time, the genetic algorithm selected features separately from the extracted features of the two models. After that, we hybridized the selected features by the serial-based approach. We classified our images into five different classes, using machine learning classifiers in the final step.

**Figure 2:**
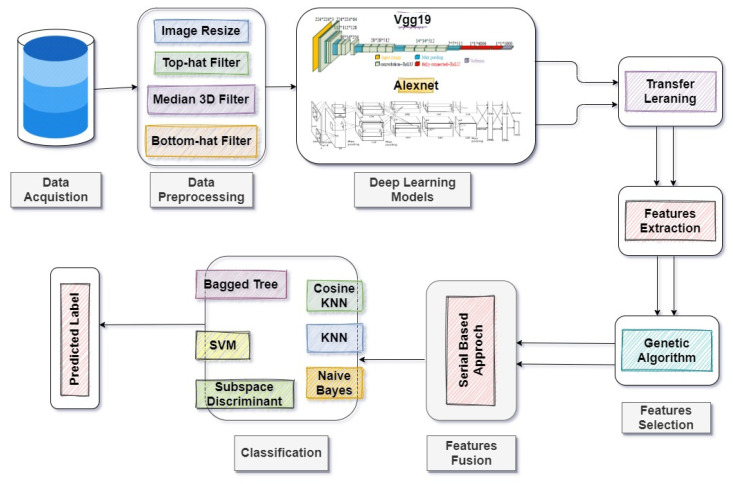
The proposed methodology for the detection and classification of stomach diseases.

To observe the performance of our models, we calculated classification results using machine learning classifiers after feature extraction and the feature selection process. To obtain the absolute accuracy of the proposed methodology, we provided the hybridized selected features to different machine learning classifiers. We observed that the proposed cubic SVM outclassed the other classifiers with an accuracy of 99.8% in the proposed methodology. The flow diagram of the proposed technique is shown in Figure 2.

### 3.1. Data Acquisition

We collected our dataset of endoscopic videos from a medical specialist in Sargodha. Initially, our dataset consisted of 118 videos of 80 patients. We selected 50 videos based on quality and converted them into frames using MATLAB [45]. After that, we labeled our dataset with the help of a medical expert and made sure the finalized dataset was in a normalized form. Our final dataset consisted of five classes. The names of the classes were gastritis, ulcer, bleeding, esophagitis, and healthy. There were 527, 519, 514, 519, and 511 images, respectively, from each class. Furthermore, we split our data into training and testing sets using a cross-validation technique with ten folds.

### 3.2. Data Preprocessing

Image processing plays a vital role in providing enhanced images with more information in medicine. The quality of images/frames is a significant factor in obtaining maximum accuracy. If an image/frame contains a blur or noise or is low in quality, it is necessary to improve the quality of image to obtain a better result. Firstly, we reduced the size of the images from 381 × 321 to 256 × 256. After that, we used some filters to enhance image quality. We used morphological top-hat and bottom-hat filters to binarize images for spot target detection. They calculated the morphological opening or closing of an image and then subtracted the obtained value from the original image [46]. The mathematical representations of the filters are shown in Equations (3) and (4).

(3) F={(x, f(x)) | x∈P, P ⊆ E2}

(4) B={(m, b(m)) | m∈S, S ⊆ E2}

Input image (*F*) and structuring element (*B*) are used above. The formulas are displayed in Equations (5) and (6).

(5) (F⊕B) (x)=sup {f (x−m)+b(m)}

(6) (F⊕B) (x)=inf {f (x+m)−b(m)}

These filters helped us extract the dark features, and we also obtained image details, such as edge, size, and surface.

As in medical imaging, the denoising of images was an essential part of the process. With denoising, we could obtain more accurate information than could be obtained from the original images. It was also essential to retain the important features as much as possible while denoising the image. Keeping this in mind, we used a median 3D filter to remove noise from the images.

Median filtering is a nonlinear method to denoise the images. It is generally utilized as it is highly successful at eliminating noise while protecting the edges of the original image. The median filter works by moving from one single pixel to another and taking the median value of neighboring pixels and replacing it with every pixel at the time of movement [28]. The mathematical expression is shown in Equations (7) and (8).

(7) Median (X)=X(k+1)=X(m); N=2k+1

(8) ½ {X(k)+X(k+1)}; N=2k

Since the median value should be the estimation of one of the pixels in the area, the median filter does not make new unreasonable pixel estimates when the filter rides an edge. Therefore, the median filter is vastly improved at safeguarding sharp edges than the mean filter.

**Figure 3:**
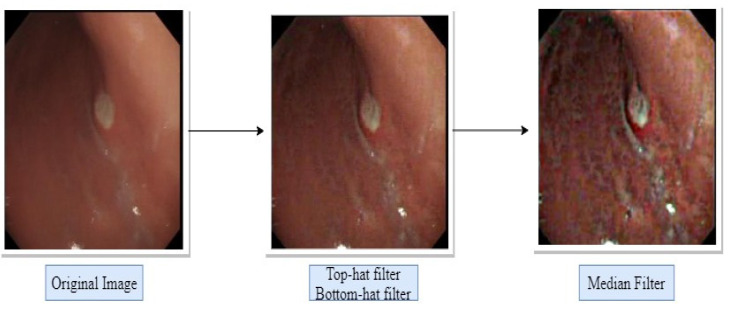
The results of preprocessing after the use of filters.

The condition of an image after every filter was applied is represented in Figure 3.

### 3.3. Feature Extraction Using Alexnet and VGG19

Feature extraction is an essential step to building a model. Several features, such as color, shape, and geometry and speeded-up robust features (SURF) can be used to develop a system. In the proposed methodology, we used transferred Alexnet for feature extraction purposes. We extracted deep features, as they have the most details, and this detail is significant in the field of medical imaging [47]. These features have details, such as color, texture, edges, etc. Color features help to calculate the evidence of the infected region. Alexnet is a CNN that consists of eight layers [23]. It used RELU instead of the tanh function, which helped it to complete training much faster than a CNN using tanh. Alexnet also provided a process for data augmentation and dropout that helped the model to reduce the overfitting problem. The standard formulas of the classification layer and the RELU layer for CNN are displayed in Equations (9) and (10).

(9) $$G\;\left[m,\;n\right]=\left(f\times h\right)\left[m,\;n\right]=\underset{j}{\sum }\underset{K}{\sum }h\;\left[j,\;k]\;f\;[m\;-\;j,\;n-k\right]$$

(10) f(x)=max(0,x)f(x)=max(0,x)

We also used VGG19 for feature extraction. VGG19 is a series of convolutional layers followed by some fully connected layers. It is a deep CNN that consists of 19 layers and was trained using the Imagenet dataset [27]. We passed the data through transferred VGG19, and all layers used three × three filters, making it small and easy to interrupt. The input layer to the last max-pooling layer model worked as a feature extractor, and the rest of the network was regarded as a classification model. After performing transfer learning, we collected our final results on an FC7 fully connected layer. The standard formulas of the batch normalization layer and the softmax layer are displayed in Equations (11) and (12).

(11) $$Batch\;Mean=\frac{1}{m}\sum_{i=1}^{m}x$$

(12) $$Softmax\;\left(x\right)i=exp\;\left(xi\right)\;/\sum_{j=1}^{n}\exp\left(xj\right)$$

### 3.4. Transfer Learning

AlexNet and VGG19 demonstrated their great characterization capacity. However, their training was time-consuming. Moreover, our dataset was not large enough to be suitable to train such a huge volume of deep networks [27]. We used transfer learning to solve this problem. We changed the last three layers of AlexNet with the following layers: a fully connected layer with five nodes, a softmax layer, and a classification layer. Furthermore, we divided our structure into two portions: the first is the pre-trained network, and the second is the transferred network. The parameters in the pre-trained network were prepared on ImageNet with a large dataset and proved effective, so these parameters needed a minor modification. The parameters used by the transferred network were a tiny part of the whole network, which is good for our small dataset compared to the vast dataset. The activation was applied to the seventh FC layer to extract features. The size of the layer that we obtained as a resultant vector of FC7 was 1 × 4096.

**Figure 4:**
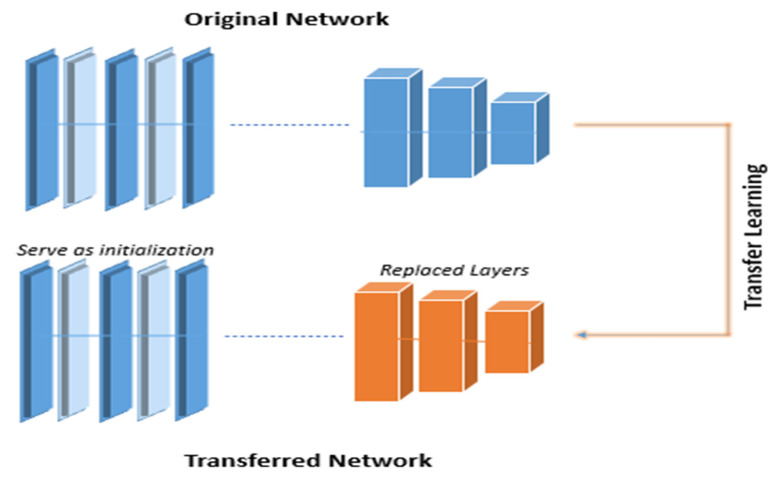
Transfer learning of networks.

The transfer learning of networks is expressed below in Figure 4.

### 3.5. Feature Selection using a Genetic Algorithm

Feature selection is an essential step in deep learning models. The accuracy of a classifier is needed to select the relevant features, and it requires much computational work. We used a genetic algorithm for feature selection, to keep these points in mind. It operated on the set of individuals, which was called population. Each generation created a new population by checking their level of fitness. We separately selected features for both models (VGG19 and Alexnet) from their extracted vectors in the proposed methodology. We used 50 chromosomes, the number of generations was 100, the crossover rate was 0.8, and the mutation rate was 0.01.

**Figure 5:**
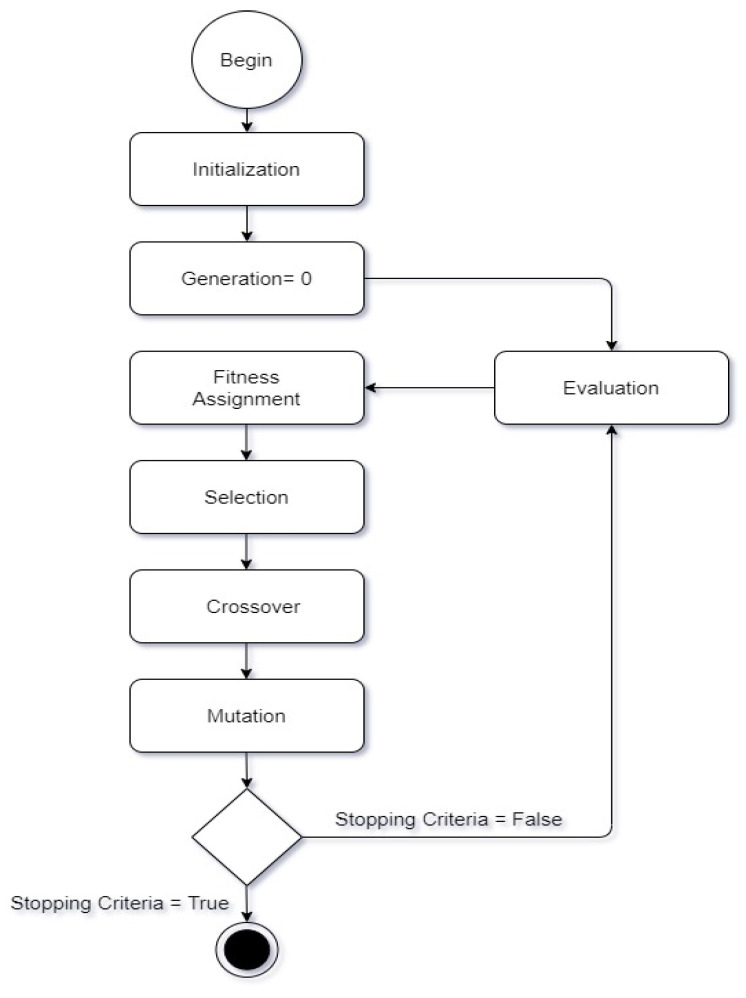
A diagram to show the process of feature selection using a genetic algorithm.

A state diagram for the selection of features with the genetic algorithm is shown in Figure 5.

### 3.6. Hybridization and fusion of features

Hybridization and fusion features are the processes where we integrated multiple features to obtain prominent features. This process also improved the discriminative power. We used the serial-based approach for this purpose. It was different from other fusion techniques as it used a complex vector rather than a super vector to combine two different features.

### 3.7. Classification

Classification is the final stage for a model where the goal is to predict the label [1]. In the proposed methodology we used the multi-class classification technique to classify the input image, based on the selected features. There are many classifiers that we used in the proposed methodology, such as decision tree, naïve Bayes, k-nearest neighbor, cubic support vector machine, bagged tree, cosine k-nearest neighbor, fine tree, and coarse tree. The proposed technique produced the best result for Cubic SVM on our dataset.

## 4. Results

### 4.1. Performance Matrix

Stomach disease detection and classification can be assessed with the help of different performance metrics. The disease classification performance measure is highly dependent on the detection rate, the ratio between the total number of pixels and infected pixels. The detection rate is also known as the probability of detection. There are some important terms, such as recall, precision, AUC, positive predictive value (PPV), negative predictive value (NPV), false positive rate (FPR), false discovery rate (FDR), and accuracy, which wereused by several researchers to analyze the stomach disease classification results [13,20,48]. There are some performance metrics given below in Table 2.

### 4.2. Feature Extraction Accuracy

In this section, we discuss the results of feature extraction using both the VGG19 and Alexnet models. We applied different machine learning classifiers, named cubic SVM, quadratic SVM, linear SVM, fine KNN, cosine KNN, fine tree, bagged tree, coarse tree, and naïve Bayes, to obtain the accuracy. We observed that cubic SVM outclassed all other classifiers, showing 99.9% accuracy, followed by fine KNN and cosine KNN with accuracies of 99.8% and 98.2%, respectively. The classification results that we obtained using VGG19 model features are displayed in Table 3.

In addition, we used Alexnet for feature extraction purposes. We applied different machine learning classifiers, named cubic SVM, quadratic SVM, linear SVM, fine KNN, cosine KNN, fine tree, bagged tree, coarse tree, and naïve Bayes, to obtain the accuracy. We observed that fine KNN showed the best result, with 99.9% accuracy, as compared to all other classifiers, followed by cubic SVM and cosine KNN, with accuracies of 99.8% and 98.2%, respectively. The precision and f1 score of fine KNN was 99.8%. Moreover, we compared the results using recall, precision, f1 score, etc. as we mentioned earlier. The results of the classification using Alexnet model features are displayed in Table 4.

### 4.3. Feature Selection Accuracy

We used a genetic algorithm for feature selection. Firstly, we applied the genetic algorithm on the extracted features of Alexnet, and after that, we applied the genetic algorithm again on the extracted features of the VGG19 model separately. We discussed the results of both models here. We applied different machine learning classifiers, named cubic SVM, quadratic SVM, linear SVM, fine KNN, cosine KNN, fine tree, bagged tree, coarse tree, and naïve Bayes, to obtain the accuracy.

We observed that fine KNN outclassed all other classifiers, showing 99.9% accuracy, followed by cubic SVM and cosine KNN, with an accuracy of 99.8% and 98.2%, respectively. Moreover, we compared the results using recall, precision, and f1 score, as we mentioned earlier. The recall and f1 score of fine KNN was 99.8%. The classification results of the genetic algorithm on Alexnet extracted features are given in Table 5.

We utilized Alexnet and VGG19 to extract features. We separately applied GA on both models to select the features from the extracted features of those two models. Here we will discuss the classification results using the GA for VGG19 model features. We observed that fine KNN outclassed all other classifiers, showing 99.8% accuracy, followed by cubic SVM and quadratic SVM with 99.7% and 99.7%, respectively. The recall and f1 scores of fine KNN were 99.8% and 99.69%, respectively. The performance of the fine tree was comparatively low compared to the other classifiers with an accuracy of 87.7%, and the recall and f1 scores were 87.4%. The results of classification using the GA for VGG19 model features are displayed in Table 6.

### 4.4. Classification Accuracy

We applied different machine learning classifiers and compared them to obtain better accuracy. The machine learning classifiers were cubic SVM, quadratic SVM, linear SVM, fine KNN, cosine KNN, fine tree, bagged tree, coarse tree, and naïve Bayes. After hybridizing our features, we gave those features to the classifiers mentioned above to obtain the best final accuracy of our model. We observed that cubic SVM outclassed all other classifiers, with 99.8% accuracy, followed by bagged tree and linear SVM, with 98.8% and 98.6%, respectively. The precision obtained using cubic SVM was 99.8%, which was 0.54% higher than the second-best classifier. The accuracy of the coarse tree was 75.9%, and coarse KNN, at 90.7%, was comparatively low compared to the other classifiers. The classification results of fused features are displayed in Table 7.

We analyzed that cubic SVM provided 100% results for every class (gastritis, ulcer, bleeding, esophagitis, and healthy) except for bleeding. In bleeding image classification, our classifiers produced 99.5% true positive results, and only 0.5% of the results were false positives. This means that 99.5% of our classifiers detected the disease correctly while only 0.5% of our classifiers produced false-positive results when there was no disease.

**Figure 6:**
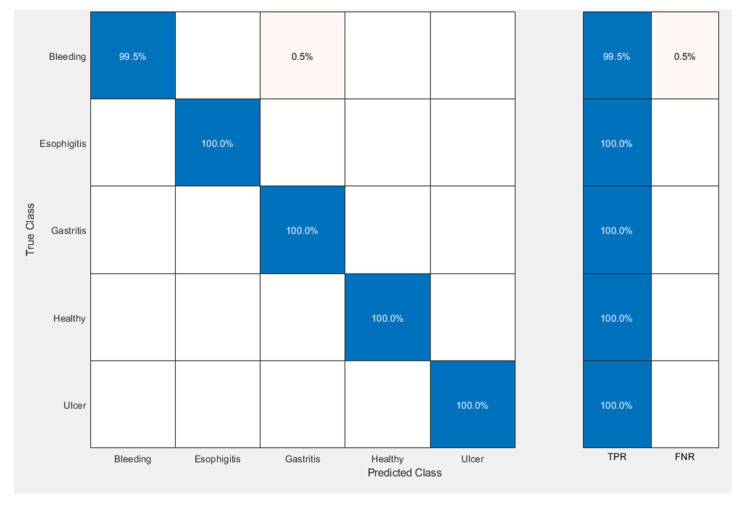
Confusion matrix (true positive rates) of cubic SVM using fused features.

The confusion matrix of cubic SVM on fused features is displayed in Figure 6.

The applied classifier Cubic SVM produced 100% results for all classes (gastritis, ulcer, bleeding, esophagitis, and healthy) when no diseases were found except for gastritis. In gastritis images, the confusion matrix shows that 99.5% was the accuracy of detection of no disease; only 0.5% of the results were false negatives. This means that our classifier wrongly predicted only 0.5% of the results when there was a disease, but our classifier judged that as no disease. So, as a result, our accuracy fell from 100% to 99.5%.

**Figure 7:**
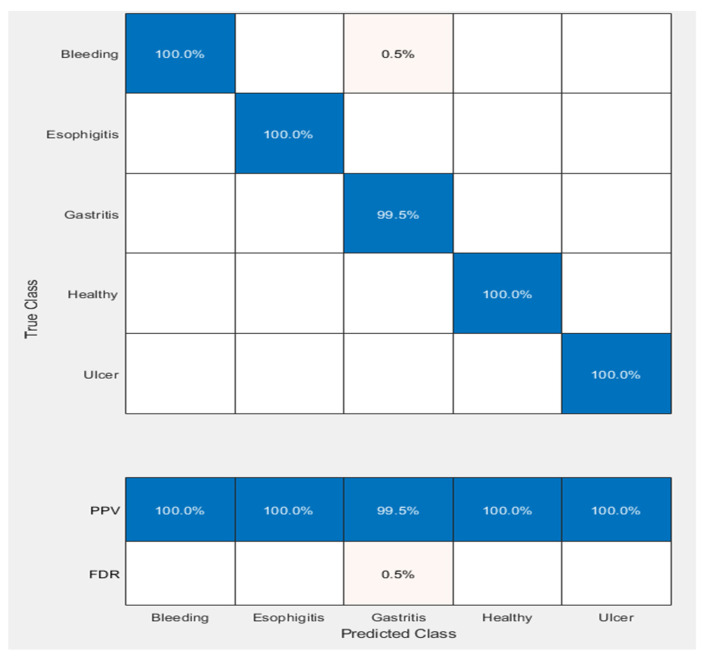
Confusion matrix (false discovery rates) of cubic SVM using fused features.

The confusion matrix of cubic SVM using fused features is displayed in Figure 7.

## 5. Discussion

A detailed discussion of the proposed methodology is described in this section. There are five major steps involved in our work, as shown in Figure 2.

The proposed methodology consists of seven steps, such as data acquisition, preprocessing, feature extraction using VGG19 and Alexnet, transfer learning, feature selection using a genetic algorithm, feature fusion, and classification. In the first step, we collected a dataset from a medical specialist. The dataset was in rough form. We normalized our collected data by taking the input of a gastrologist. In the second step, we applied different filters to our collected dataset, which was now available in images. After that, we selected two convolutional neural networks, named Alexnet and VGG19, to extract features. The best thing about these models was that there was no need to develop a system from scratch, as both models were already trained on thousands of imagenet datasets and could classify into 1000 different classes. We performed transfer learning on these models that helped in the modification process. After transfer learning, our models were modified and were ready to extract the features of our dataset.

We used the genetic algorithm for feature selection purposes. As we extracted features with two models, we needed to select features separately. One at a time, the genetic algorithm selected features separately from the extracted features of the two models. After that, we hybridized the selected features by the serial-based approach. We classified our images into five different classes using machine learning classifiers in the final step.

To observe the performance of our models, we calculated classification results using machine learning classifiers after feature extraction and the feature selection process. To obtain the final accuracy of the proposed methodology, we provided the hybridized selected features to different machine learning classifiers. The analysis of these classifiers was conducted through well-known performance metrics, including sensitivity, precision, accuracy, f1 score, true-negative rate (TNR), true-positive rate (TPR), false-negative rates (FNR), and false-positive rates (FPR). We observed that the proposed cubic SVM outclassed the other classifiers, with an accuracy of 99.8%.

We also provided the same dataset that we used in the proposed methodology to the pre-trained Alexnet and VGG19 models for the sake of comparison. We observed that the Alexnet model produced better results than the VGG19 model for our dataset. The obtained accuracies on our dataset were 87.7% for Alexnet and 86.4 % for VGG19. The results were relatively low compared to the proposed hybrid approach that produced 99.8% results on the same dataset.

In the end, we compared our results with the existing state-of-the-art techniques, in terms of accuracies. We concluded that the proposed hybrid technique helped improve the results. The comparison is shown in Table 8.

## 6. Conclusions and Future Work

Manual classification of endoscopic lesions in the stomach is a challenging task, and there is a need to have CAD for efficient and accurate results. This paper proposed a deep-learning-based model following the feature fusion framework to classify four different stomach diseases. We selected two different CNN models (VGG19 and Alexnet) to extract features and then used transfer learning on the feature vectors before using them as feature extractors. In feature selection, a heuristic GA selected rich information from the extracted feature vectors. The hybridization helped to improve the accuracy of the proposed model, due to strong predictor values. We obtained 99.8% accuracy using the cubic SVM classifier on the given dataset. The proposed framework selected only 50% of the best features, which is a limiting aspect and can be tackled in future work. Furthermore, a study can be performed compare the histopathological aspect of the lesion after numerous comparisons of the processed images with the microscopic images. This method could be credible for medical practice.

## Figures and Tables

**Table 1:** Existing studies on stomach disease detection and classification.

Techniques/Methods	Disease	Dataset	Accuracy	Reference
Inception V3 and SVM	Bleeding	2352 Images	97.71%	[37]
CNN	Gastric Cancer	2434 Images	95%	[38]
VGG16 and SVM	Ulcer and Bleeding	6000 Images	98.4%	[36]
CNN	Gastric Cancer	2088 Images	90.91%	[14]
CNN	Ulcer	17640 Images	96.7%	[39]
CNN	Gastritis	5470 Images	88.15%	[40]
DCNN	Gastric Cancer	763 Images	96%	[41]
ResNet and LSTM	Ulcer and Crohn’s	52,471 Frames	97.05%	[29]
SVM and RF	Stomach cancer	3106 Images	96.36%	[42]
CNN	Gastric Cancer	13,584 Images	92.2%	[10]
CNN	Gastric Cancer	1000 Images	87.6%	[6]
DFT and NB	Stomach Cancer	900 Images	90.27%	[33]
SVM and MLP	Colon Abnormalities	1670 Images	96.5%	[32]
CNN	Gastric Cancer	3257 Images	96.88%	[25]
ANN, RF, LBP, and HOG	Stomach Cancer	180 Images	96.29%	[22]
SVM	Gastric Cancer	207 Images	96.3%	[20]
ANN	Stomach Cancer	270 Images	88.9%	[11]
SVM and MLP	Ulcer	2333 Images	94.07%	[17]
BPNN	Stomach Disorder	40 Images	87.5%	[1]
ESD	Gastric Neoplasms	1052 Patients	93.3%	[43]
BPNN	Crohn’s Disease	387 Patients	97.67%	[18]
ME-NBI	Gastric Cancer	76 Patients	81.6%	[44]
SVM	Gastrointestinal Hemorrhage	2920 Images	98.95%	[21]
CNN and ELM	Digestion Disease	25 Examinations	97.25%	[12]

**Table 2:** Performance matrices.

Sr. No	Performance Matrix	Formula	Description
1.	Recall	Re=PtPt+Nf	Where, Pt = True Positives Pf = False positives Nf = False Negatives Nt = True Negatives
2.	Precision	$\mathrm{Pr}=\frac{\mathrm{Nt}}{\mathrm{Nt}+\mathrm{Pf}}$	
3.	PPV	$\mathrm{PPV}=\frac{\mathrm{Pt}}{\mathrm{Pt}+\mathrm{Pf}}$	
4.	NPV	$\mathrm{NPV}=\frac{\mathrm{Nt}}{\mathrm{Nt}+\mathrm{Nf}}$	
5.	FPR	$\mathrm{FPR}=\frac{\mathrm{Pf}}{\mathrm{Pf}+\mathrm{Nt}}$	
6.	FDR	$\mathrm{FDR}=\frac{\mathrm{Pf}}{\mathrm{Pf}+\mathrm{Pt}}$	

**Table 3:** Classification results using VGG19 model features.

Classifier	Recall (%)	Precision (%)	F1 Score (%)	FPR	AUC	Accuracy (%)	FNR (%)
Fine Tree	88.6	88.8	88.7	0.028	0.946	88.7	11.4
Cubic SVM	99.8	99.8	99.8	0	1	99.9	0.2
Fine KNN	99.6	99.6	99.6	0	1	99.8	0.4
Cosine KNN	98.2	98.2	98.2	0.006	1	98.2	1.8
Bagged Tree	97.8	97.2	97.4	0.006	1	97.2	2.2
Linear SVM	97.4	96.6	96.9	0.01	1	96.6	2.6
Coarse Tree	73	63.6	67.9	0.068	0.91	73.1	27

**Table 4:** Classification results using Alexnet model features.

Classifier	Recall (%)	Precision (%)	F1 Score (%)	FPR	AUC	Accuracy (%)	FNR (%)
Fine Tree	92.4	92.8	92.59	0.0016	0.964	92.7	7.6
Cubic SVM	99.6	99.6	99.6	0	1	99.8	0.4
Fine KNN	99.8	99.8	99.8	0	1	99.9	0.2
Cosine KNN	98.2	98	98.1	0.004	1	98.2	1.8
Bagged Tree	98	98	98	0.002	1	98.1	2
Linear SVM	98.2	97.2	97.69	0.006	1	97.2	1.8
Coarse KNN	86.4	86.6	86.5	0.034	0.964	86.4	13.6

**Table 5:** Classification results using GA for Alexnet model features.

Classifier	Recall (%)	Precision (%)	F1 Score (%)	FPR	AUC	Accuracy (%)	FNR (%)
Fine Tree	90.8	91	90.89	0.022	0.954	90.9	9.2
Cubic SVM	99.6	99.6	99.80	0	1	99.8	0.4
Fine KNN	99.8	99.8	99.8	0	1	99.9	0.2
Cosine KNN	98.4	98	97.60	0.004	1	98.2	1.6
Bagged Tree	97.4	97.4	97.56	0.006	1	97.3	2.6
Linear SVM	96.6	96.8	99.69	0.008	1	96.7	3.4
Quadratic SVM	99.6	99.6	99.8	0	1	99.8	0.4

**Table 6:** Classification results using the GA for VGG19 model features.

Classifier	Recall (%)	Precision (%)	F1 Score (%)	FPR	AUC	Accuracy (%)	FNR (%)
Fine Tree	87.4	87.6	87.49	0.03	0.742	87.7	12.6
Cubic SVM	99.4	99.6	99.49	0	1	99.7	0.6
Fine KNN	99.8	99.6	99.69	0	0.99	99.8	0.2
Cosine KNN	98.6	98.4	98.49	0.004	1	98.5	1.4
Bagged Tree	97.2	97.2	97.2	0.004	1	97.3	2.8
Linear SVM	97.6	97.4	99.49	0.006	1	97.6	2.4
Quadratic SVM	99.6	99.6	99.6	0	1	99.7	0.4

**Table 7:** Classification results of fused features.

Classifier	Recall (%)	Precision (%)	F1 Score (%)	FPR	AUC	Accuracy (%)	FNR (%)
Fine Tree	90.33	90.43	90.34	0.024	0.962	90.3	9.67
Cosine KNN	99.26	99.26	99.26	0.002	1	99.3	0.74
Bagged Tree	98.84	98.84	98.84	0.004	1	98.8	1.16
Linear SVM	98.62	98.64	98.63	0.004	1	98.6	1.38
Coarse Tree	75.84	79.74	77.74	0.06	0.914	75.9	24.16
Cubic SVM	99.8	99.8	99.8	0	1	99.8	0.2
Naïve Bayes	96.16	96.24	96.19	0.008	0.976	96.2	3.84
Coarse KNN	90.74	91.6	91.16	0.024	0.98	90.7	9.26

**Table 8:** Comparison of the proposed methodology with the state-of-the-art approaches.

Author/Year	Techniques/Methods	Disease	Dataset	Results
[39]	CNN	Ulcer	17,640 Images	96.7%
[41]	DCNN	Gastric Cancer	763 Images	96%
[36]	VGG16 and SVM	Ulcer	6000 Images	98.4%
[37]	Inception V3 and SVM	Bleeding	2352 Images	97.71%
Proposed Methodology	VGG19, Alexnet, and Cubic SVM	Ulcer, Bleeding, Esophagitis, and Gastritis	2600 Images	99.8%

## Data Availability

Not Applicable.
